# Therapeutic Parent–Child Communication and Health Outcomes in the Childhood Cancer Context: A Scoping Review

**DOI:** 10.3390/cancers16112152

**Published:** 2024-06-06

**Authors:** Heeyeon Son, Nani Kim

**Affiliations:** 1 College of Nursing, University of Tennessee, 1412 Circle Drive, Knoxville, TN 37996, USA; 2 School of Nursing, The University of Texas at Austin, 1710 Red River St., Austin, TX 78712, USA; nanikim@utexas.edu

**Keywords:** child, adolescent, cancer, communication, health outcomes

## Abstract

**Simple Summary:**

Family communication in the cancer context has gained attention as a crucial factor related to the quality of life of children and adolescents. The aim of this review was to examine therapeutic parent–child communication and its impact on health outcomes in children and adolescents with cancer. This review identified an emerging body of evidence that therapeutic parent–child communication contributes to better psychological health outcomes at both the individual and family levels in the childhood cancer context. A comprehensive understanding of the impact of family communication may provide knowledge for developing interventions to improve family communication and health outcomes.

**Abstract:**

Family communication has been thought to be an important area to support children’s adjustment to a cancer diagnosis. However, the characteristics of therapeutic parent–child communication that contribute to better patient outcomes and the specific patient health outcomes have been less explored. This current review explored the characteristics of therapeutic parent–child communication and its physical and psychological health outcomes. A total of 5034 articles were initially identified, and only 10 articles remained for inclusion in this review after application of the exclusion criteria. Most studies used a cross-sectional design and measured verbal communication characteristics and its psychological outcomes, but no physical outcomes. The characteristics of therapeutic verbal communication (openness, maternal validation, quality of information shared, etc.) and nonverbal communication (eye contact, close physical distance, and acknowledging behaviors) were identified. The psychological health outcomes included less distress, a lower level of PTSS, less internalizing and externalizing of symptoms, increased levels of social emotional competencies, better peer relationships, and more cooperation during the procedure at the individual level. Increased family cohesion and family adaptation were family-level outcomes. Longitudinal studies are needed to identify what qualities of communication predict better psychological outcomes so that interventions can be developed and tested. In addition, physical outcomes should be evaluated.

## 1. Introduction

Recently, due to advances in technology, early diagnosis, and treatment, up to 85% of childhood cancer patients look forward to long-term survival [1]. This improvement in survival makes childhood cancer a chronic illness with long-term sequelae from treatment [2] and adds an important component to cancer care of focusing on quality of, life which includes a sense of well-being and coping for cancer patients and their families [3].

Family support is among the diverse factors found to contribute to better quality of life [4,5,6]. Research has described the importance of the role of family in dealing with cancer-related distress and adjusting well to the cancer diagnosis by overcoming psychological challenges such as internalizing and externalizing behaviors, as well as anxiety in the childhood cancer context [7]. Without proper support, children and adolescents with cancer can be at risk of maladjustment to a cancer diagnosis [7,8], and parents may struggle to provide optimal care due to unresolved stress and fear related to uncertainty [9].

Among the diverse roles that family plays in supporting children and adolescents along the childhood cancer journey, communication has been found to be a potentially important area [10]. Communication is an indication of the quality of dyadic interactions, including the level of intimacy, trust, or conflict. By communicating, parents can share information, offer support, and socialize their children. In the childhood cancer context, family communication is defined as family members’ ability to communicate and express their thoughts and feelings in the midst of a stressful situation [11]. Specifically, the ability to share emotional needs has been reported as essential for the development of healthy coping strategies in children and adolescents [12,13].

The relationship between effective family communication and coping or adjustment is well supported in the context of adults’ chronic illnesses, including cancer [14,15]. When patients could share their emotions with their family members, they were less likely to experience disease related distress [16]. Positive family communication between the patient and caregiver mediated the relationship between family hardiness, caregiver positivity [17], and reduced caregiver burden [18]. When family members and patients shared all cancer-related information, all were more likely to experience overall adjustment and higher rates of psychological well-being [19,20]. Family members also benefit from the open communication that involves sharing emotions and emotional support [21,22,23]. Other benefits from family communication include fewer conflicts between family and physician and improved decision making [24,25]. The association between parent–child communication characterized by the openness of emotions and adjustment is also widely accepted in the childhood cancer context [26]. For example, by communicating with their parents, children with cancer were well informed about their condition and showed psychological stability and better adjustment and resilience [27,28].

Despite the evidence demonstrating the importance of communication, there are several gaps that need to be addressed in order to develop and test potentially effective interventions to improve the communication between parents and children/adolescents with cancer. The most important gap is a lack of knowledge around the characteristics of parent–child communication that are associated with better patient outcomes in terms of both psychological and physical outcomes. In addition, the expected benefits other than coping and adjustment have not been fully explored as much as they have been in the adult cancer context. Thus, the purpose of this paper was to explore the characteristics of parent–child communication that result in better physical or psychological patient outcomes in the childhood cancer context.

## 2. Materials and Methods

This literature review was conducted in accordance with the methodological guidelines outlined in the Principles of the Preferred Reporting Items for Systematic Reviews and Meta-Analysis Extension for Scoping Reviews (PRISMA-ScR) for data extraction and reporting [29]. A scoping review, which falls under the category of systematic literature reviews, delineates the extent of existing research evidence across diverse methods or disciplines. This methodology fit the purpose of this review in that we wanted to understand the breadth of the evidence and highlight the gaps as well as offer recommendations for potential future investigations [29,30].

### 2.1. Literature Search and Screening

A health sciences librarian with expertise in systematic review methodologies assisted with the selection of databases and the development of search terms. References were obtained by searching in PubMed, CINAHL PsycINFO, and Scopus using the MeSH terms based on keywords, including ‘parent-child’, ‘parent-adolescent’, ‘communication’, and ‘malignancy.’ The MeSH terms based on each keyword were generated by a health sciences librarian for each database. The search process was conducted from December 2022 to December 2023.

Two reviewers (HS and NK) independently screened the titles and abstracts of the studies obtained through the databases to determine their eligibility for full-text review. The studies included were selected based on the following criteria: (a) written in English and peer-reviewed, (b) published after January 2014, (c) the target population was children and adolescents aged up to 24 years with any type of cancer, and (d) the inclusion of physical or psychological outcomes. Nonresearch articles, such as literature reviews, letters, editorials, dissertations, and opinion pieces, were excluded. Also, studies focusing on disclosing children’s cancer diagnoses only were excluded, as the scope of our topic was broader than disclosing the diagnosis. Finally, any studies focused on the effectiveness of the interventions or the validity of psychological measurements were not included. Following the screening process, the two reviewers discussed conflicts in the eligibility, and a consensus was established.

### 2.2. Data Extraction

The studies selected beyond the initial screening were divided between the two reviewers. Each reviewer read the full-text articles and had discussions with each other to determine whether the articles satisfied the inclusion and exclusion criteria, as well as the strengths and weaknesses of each study to be included. The two reviewers independently completed an investigator-developed table, which integrated the characteristics of parent–child/adolescent communication and the relationships between the communication and health outcomes. The first author subsequently reviewed the table to verify the accuracy of the data extraction process. Discrepancies regarding the decision on which literature should be included were resolved at regular meetings. In this scoping review, a quality assessment score was not determined; rather, we employed critical appraisal to scrutinize the strength and limitations of each study. This approach enabled us to encompass diverse sources of evidence on our broad topic.

## 3. Results

**Figure 1:**
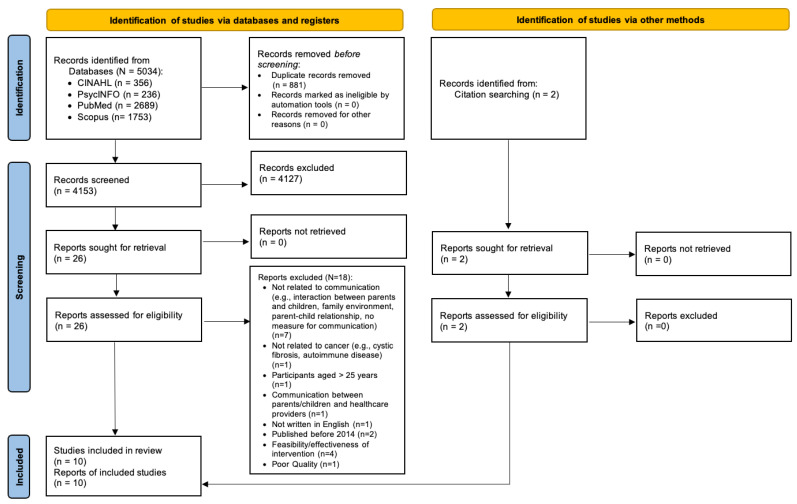
PRISMA flow diagram of study selection [31].

A total of 5034 articles were identified through database searches. After removing duplicates, 4153 remained. An additional two studies were identified through manual searching reference lists. Abstract and title screening removed 4127 nonrelevant articles, and full-text review removed an additional 18 studies. Out of the 18 articles excluded, 1 article was excluded because of its irrelevance to our research questions and its low quality as evidenced by several errors in text, inconsistency between research questions and findings, and statistical tests. One additional article was excluded because it mainly focused on measuring the parent–child relationship without including the parent–child communication component. As a result, 10 studies were retained. Figure 1 provides our PRISMA flow diagram [31].

### 3.1. Overview of Included Studies

The included studies spanned quantitative (90%, *n* = 9) and a mixed-method design (*n* = 1). Among the quantitative studies, the majority used a cross-sectional, correlational study design (70%, *n* = 7), with only three using a longitudinal study design. Methodological elements, sample demographics, and other characteristics are summarized in Table 1.

### 3.2. The Characteristics of Children and Adolescents with Cancer

The age range of patients was broad, from 3 to 24 years old, with a majority of the participants identifying as White [8,26,32,33,34,35]. Nine out of ten studies provided demographic information about child/adolescents’ biological sex [8,26,32,33,34,35,36,37,38]. The majority of studies included a slightly higher number of male participants [32,33,35,36,37]. However, no influence of sex differences on parent–child communication was reported.

Overall, the participants in the included studies reported diverse types and statuses of cancer diagnoses. Studies mostly included patients who were newly diagnosed with cancer, based on the timeframe ranging from 1 to 18 months from the initial diagnosis [8,33,36,37]. Cancer-related communication was assessed among study participants undergoing cancer treatment in six articles [8,26,33,35,36,37]. One article provided detailed information regarding the types of cancer and progress (advanced vs. nonadvanced cancer), as well as whether the diagnosis was relapsed or refractory [26]. Four articles identified the types of treatments participants received, such as chemotherapy, surgery, and radiation [8,32,34,36], whereas the remaining six articles did not provide this information [26,33,35,37,38,39].

It was notable that one article examined illness-related factors, such as years since diagnosis and relapse status, as potential influences on communication. Tillery et al. [34] examined the influence of the time since diagnosis and relapse status on the parent–child relationship, which included parent–child communication practices. An increased time since diagnosis was related to a higher chance of being either struggling (lower than average levels of parent–child relationship) or normative (average levels of parent–child relationship) groups. When children had a relapsed cancer diagnosis, their families were more likely to have a highly involved parent–child relationship, which was characterized by reports of above-average levels of parent–child communication.

**Table 1:** Summary of studies included in this review.

Study/ Country	Objective	Design	Sample and Age Range (Groups)	Independent Variable (Measurement and Method)	Dependent Variable (Measurement and Method)	Relationship/ Characteristics of PAC
Greeff et al. [38]/ Belgium	Identify resilience factors associated with family adaptation	Cross-sectional, correlational	26 parents and 25 children aged 12–24 (G3 and 4)	Parents’ and children’s self-report of family communication (affirming/incendiary communication) (FPSC)	Parents’ and children’s self-report of family adaptation (FACI8)	(1) Positive correlation between affirming communication and family adaptation in the reports from parents (*r*_s_ = 0.62) and children (*r*_s_ = 0.71) (2) Negative correlation between incendiary communication and family adaptation in the reports from children (*r*_s_ = −0.59) (3) Family problem solving communication found to be the predictor of family adaptation in the reports from parents (*b* = 0.65) and children (*b* = 0.52)
Phillips-Salimi et al. [33]. USA	Identify the relationships among adolescents’ and parents’ perceptions on communication, family adaptability, and cohesion	Cross-sectional, correlational	70 dyads: AYA aged 11–19 (G3&4)	Adolescents’ and parents’ self-reports on perceptions of (a) open family communication, and (b) problems in family communication (PACS)	Identify the relationships among adolescents’ and parents’ perceptions on communication, family adaptability, and cohesion (FACES-II)	Controlling for age and sex of AYA and parents, (1) Four predictors found for adolescent-perceived communication: (a) adolescent-perceived family adaptability (*^®^β* = 0.49), (b) adolescent–family cohesion (*β* = 0.67), (c) parent–family adaptability (*β* = 0.33), and (d) parent–family cohesion (*β* = 0.40) (2) Four predictors found for parent-perceived communication: (a) adolescent-perceived family adaptability (*β* = 0.34), (b) adolescent–family cohesion (*β* = 0.33), (c) parent–family adaptability (*β* = 0.37), and (d) parent–family cohesion (*β* = 0.43)
Murphy et al. [8]/ USA	Examine potential risk factors for adolescent PTSS at T1 (2 months after diagnosis), T2 (3 months after T2), and T3 (12-month follow-up)	Longitudinal, nonexperimental	41 dyads: Adolescents aged 5–17 (G2, 3, and 4)	Observed maternal communication: macro level at T1 (IFIRS): harsh communication and withdrawn communication; observed maternal communication: micro level at T2 (IFIRS): solicit and validation	Adolescents’ and maternal self-report of the PTSS (the Impact of Events Scale–Revised) at T1 and T3	(1) No relationships between maternal harsh/withdrawn communication and adolescent PTSS (2) The indirect effect of maternal PTSS on adolescent PTSS through maternal validation (*b* = 0.01, kappa^2^ = 0.10) (3) Controlling for adolescent PTSS, two predictors found for adolescent PTSS: (a) adolescent PTSS (*β* = 0.37) and (b) maternal validation (*β* = −0.32)
Bai et al. [36]/ USA	Examine the associations between parent interaction behaviors, parent distress, child distress, and child cooperation during cancer-related port access placement across timepoints (T1–T4)	Longitudinal, nonexperimental	43 dyads: Children aged 3–12 (G2 and 3)	Observation of parent caring verbal/nonverbal interactions: caring parent verbal interaction (P-CaReSS) and nonverbal behaviors (duration)	Observations of (a) child distress, (b) parent/child distress, and (c) child cooperation: (1) verbal/nonverbal child distress (the Karmanos Child Coping and Distress scale), (2) Parent/child distress (the Wong–Baker Faces Scale), (3) Child cooperation (CCS)	Children’s low verbal/nonverbal distress found following parents’ caring behaviors (eye contact, comforting, supporting/allowing, less availability, verbal protecting, avoiding assumption, believing in/esteem), except for verbal forms of care (e.g., criticizing, apologizing) (Yule’s Q ranged from −0.85 to −0.99)
Keim et al. [26]/ USA	Examine the relationships between PAC and adjustment at T1 (enrolment) and T2 (one year later)	Longitudinal, nonexperimental	55 children with advanced cancer; 70 with nonadvanced disease; 60 without cancer as the control group and their mothers: adolescents aged 10–17 (G3 and 4)	Children’s self-reports on communication with their mother and father, separately (PACS)	Mothers’ self-reports on (a) child adjustment, (b) anxious/depressed scores, and (c) withdrawn/depressed scores (the Child Behavior Checklist)	The relationship between parent–child communication at T1 and child adjustment at T2: (1) Higher maternal openness in communication at T1 predicted lower withdrawn/depressed scores for children with advanced cancer at T2 (*b* = −0.14, *t* = 2.82) (2) Fewer problems in communication with mothers at T1 predicted lower withdrawn/depressed scores for children with advanced cancer at T2 (*b* = 0.14, *t* = 3.47) (3) Higher openness in communication with fathers at T1 predicted lower anxious/depressed (*b* = −0.12, *t* = 2.51) and withdrawn/depressed scores (*b* = −0.10, *t* = 2.80) at T2 in advanced cancer
Viola et al. [35]/ USA	Examine associations among problem-solving skills, PAC, parent–adolescent dyadic functioning, and distress	Cross-sectional, correlational	39 dyads: Adolescents aged 14–20 (G4)	Parents’ and adolescents’ self-reports of parent-adolescent cancer-related communication (CRCP)	Adolescents’ self-report of the level of adolescent distress (BSI)	No significant relationship between adolescent-reported cancer related communication problems and adolescents’ distress
Tillery et al. [34]/ USA	Identify the relationships between PAC and psychosocial outcomes	Cross-sectional, correlational	165 dyads: adolescents aged 10–19 (G3 and 4)	Parents’ self-report of the parent–child relationship quality (PRQ): involvement, attachment, communication (quality of information exchange), parenting confidence, relational frustration	Children’s self-report of psychosocial outcomes: (1) post-traumatic stress symptoms (22-item UCLA PTSD Reaction Index for DSM-IV), (2) internalizing difficulties (BASC-2), (3) social functioning (self-regulation, empathy, responsibility, and social competence (SEARS)	Adolescents of caregivers who reported struggling relationship patterns (below average levels of parent–child relationship functioning across several domains) were more likely to report (1) increased level of PTSS (χ^2^ = 35.06), (2) elevated levels of internalizing symptoms (χ^2^ = 10.62), and (3) poorer social functioning (χ^2^ = 16.38) compared to youth of caregivers who reported normative or above average levels of relationship function
Al Ghriwati et al. [32]/ USA	Identify subtypes of family relationships and the effects of relationships on child adjustment upon treatment completion within 7 months	Secondary analysis, longitudinal data	81 dyads: Children aged 6–14 (G3 and 4)	Children’s self-report of (1) closeness (e.g., how frequently you share information about things that you want others to know) and (2) discord (e.g., how frequently you disagree and quarrel with others) (NRI-RQV)	Caregivers’ self-report of children’s status: (1) internalizing and externalizing symptoms (CBCL), (2) peer relationships (PROMIS), (3) family functioning (FAD-GFS), (4) quality of life (the Pediatric Quality of Life Inventory 4.0)	(1) Children from families characterized by low closeness/high discord reported significantly greater difficulties with peer relationships (χ^2^ = 4.12) and higher externalizing symptoms (χ^2^ = 5.68) than those with high closeness/low discord in their families (2) Children from families characterized by low closeness/high sibling-only discord reported significantly poor social functioning than those from high closeness/low discord families (χ^2^ = 3.99)
Barrios et al. [39]/ Spain	Explore (1) the openness about cancer, (2) the relationships between the types of communication and children’s emotion	Cross-sectional, mixed method	52 dyads: children aged 6–14 (G3 and 4)	Self-report of open communication: the degree of information given to the child as categorized by (1) direct honest information, (2) nuanced or distorted information, and (3) no information at all	Self-report of child’s emotion (e.g., fear, anger, sadness, happiness, pain, boredom, loneliness, shame) and mother’s subjective emotion (e.g., fear, anger, sadness, frustration, anxiety, guilt) during the qualitative interviews	(1) Truly informed children were much less likely to express fear (*p* = 0.011) (2) The willingness to communicate with parents was higher in children whose mothers did not express anxiety (*p* = 0.003)
Park et al. [37]/ Republic of Korea	Identify risk and protective factors for family resilience that affect the adaptation of families of children with cancer	Cross-sectional, correlational	111 dyads: children’s mean age of 8.3 (N/A)	Parents’ self-report of family communication (the Family Problem-Solving Communication Scale)	Parents’ self-report of family resilience (adaptation) (APGAR questionnaire)	Family communication skills found to be predictive of family adaptation (*β =* 0.40)

PAC, parent–adolescent communication; FPSC, Family Problem Solving Communication; FACI8, the Family Attachment and Changeability Index 8; AYA, adolescents and young adults; PACS, Parent Adolescent Communication Scale; FACES-II, Family Adaptability and Cohesion Evaluation Scales; T1, time 1; T2, time 2; T3, time 3; T4, time 4; IFIRS, he Iowa Family Interaction Scale; PTSS, post-traumatic stress symptoms; P-CaReSS, Parent Caring Response Scoring System; CCS, Child Cooperation Scale; CRCP, Cancer-related Communication Problems Scale; BSI, Global Severity Index of the Brief Symptom Inventory; PRQ, Parenting Relationship Questionnaire; PTSD, post-traumatic stress disorder; BASC-2, Behavior Assessment System for Children, Secondary Edition; SEARS, Social and Emotional Assets and Resilience Scale; NRI-RQV, modified network of Relationship Inventory-Relationship Qualities Version; CBCL, Childhood Behavior Checklist; PROMIS, Patient-Reported Outcome Measure Information System; FAD-GFS, Family Assessment Device-General Functioning Scale; APGAR, Family Adaptability, Partnership, Growth, Affection, and Resolve; G1, infants (~1 year); G2, toddlers/preschoolers (2–5 years); G3, school age (6–12 years), G4, adolescence (13–24 years).

### 3.3. The Characteristics of Parents of Children or Adolescents with Cancer

Seven out of ten studies included a self-report from either the mothers or fathers of children or adolescents with cancer [32,33,34,35,36,37,38]. In the case of proxy reports, the majority of reporters were mothers [8,26,34,35,37,38,39]. However, none of the included studies provided information regarding the possible influence of parents’ sex on communication practice. It is noteworthy that two studies addressed the differences in communication between mothers and fathers [26,33]. In one of the studies, children’s reports on mothers’ and fathers’ communication were analyzed separately, indicating that fathers’ patterns of openness and problems in communication were similar to mothers’ communication patterns [26]. In another study, only the parent corresponding to the biological sex of the patient was included for analysis [33].

Most articles provided demographic information about parents’ sex and age range, but limited demographic information was provided. Regarding parents’ marital status, only three articles indicated that a majority of parents were married [32,37,38]. None of the studies included marital status as a factor in assessing its potential influence on parent–child communication.

### 3.4. Parent–Child Communication in the Childhood Cancer Context

#### 3.4.1. Types of Communication Being Measured

Each of the 10 studies used a different definition of parent–child communication and measured it differently. A majority of studies (*n* = 8) focused on measuring verbal communication only [26,32,33,34,35,37,38,39], while two studies measured both verbal and nonverbal communication [8,36].

The verbal communication measured included the degree of openness of the parent–child communication [26,33,39], the attitude of the persons communicating [32,37,38], or both the attitude and amount of information shared during the communication [34,35]. For example, the degree of openness of communication was assessed based on whether they communicated about their disease or emotions without holding back or had nondistorted communication [26,33,39]. In the articles retained, the attitude during the communication was described as either affirming or incendiary [38] using the frequency of agreement or disagreement [32], and the parent–child relationship was described by the level of involvement or attachment, the amount of information sharing that was present, parenting confidence, and relational confidence [34].

Two studies measured both verbal and nonverbal communication. For example, Bai et al. [36] measured the presence or absence of caring verbal and nonverbal interactions during port insertion, and Murphy et al. [8] observed maternal communication (i.e., whether it was harsh or withdrawn at the macro level) and whether maternal communication involved solicitation, which was defined as questions asked by mothers to elicit children’s responses or validation at the microlevel.

#### 3.4.2. Types of Communication Being Measured

The majority of studies relied on self-report measures of children, adolescents, and their parents (proxy) [26,32,33,34,35,38,39]. Three studies included both parents’ and children’s reports of their parent–child communication practices by having participants complete the same measures, which included the Parent-Adolescent Communication Scale (PACS) to assess openness and problems in parent–child communication, the Family Problem Solving Communication (FPSC) to assess affirming and incendiary communication, and the Cancer-related Communication Scale (CRCP) to measure cancer-related communication problems [33,35,38]. In the rest of the studies, the children/adolescents or parents were asked to report parent–child communication characteristics using different measurements, such as closeness and discord in communication, using the subscale of the modified Network of Relationship Inventory-Relationship Qualities Version (NRI-RQV) [32], children’s report of their parents’ openness and problems in communication practices using PACS [26], and parent reports of closeness and discord in communication using the Parent–Child Relationship Quality Questionnaire (PRQ) subscales on attachment and communication [34]. In one mixed-design study, the types of parent–child communication were assessed through parent interviews and coded as three types of communication including direct, honest information, nuanced or distorted information, and no information [39]. All of the measures were reliable and psychometrically valid. The Cronbach’s alpha of reliability ranged from 0.83 to 0.94 in each study, except the CRCP, with a Cronbach’s alpha of 0.69 (adolescent) and 0.61 (parents), which was originally developed to measure cancer-related communication problems between partners in the adult cancer context [40].

Among the 10 studies, only 2 studies involved observations performed by trained research assistants [8,36]. In one observation study, Bai et al. [36] used the Parent Caring Response Scoring System (PCaReSS) to measure the presence or absence of caring verbal and nonverbal communication during port insertion, with an 80% agreement between the trained coders. The second observation study by Murphy et al. [8] used the Iowa Family Interaction Scale (IFIRS) to measure harsh or withdrawn communication or communication involving either solicitation or validation, demonstrating an inter-rater reliability of 80%, with average intraclass correlations exceeding 0.60.

### 3.5. The Influence of Age and Developmental Stage

Among the diverse demographic variables that could possibly impact parent–child communication practices, age and the developmental stage were examined in this literature review due to the inclusion of a wide range of age groups. We found that the age group of study participants in this scoping review was broad, ranging from 3 (toddler) to 24 (late adolescence). One out of ten articles did not provide specific information regarding participants’ age range but only the mean. Among the articles that provided the age range, the broadest one included participants aged between 3 and 12 years (from toddler to adolescence) [36]. Despite the broad age range, the influence of age was not the focus of any of the studies included. However, few studies examined or considered the influence of age on communication practices. For example, Murphy et al. [8] found that harsh maternal communication was negatively related to adolescent age, suggesting that mothers of older adolescents demonstrated less irritability and frustration with their children during communication. Phillips-Salimi et al. [33] recognized the influence of age on parent–child communication and controlled for it in a statistical analysis of the relationships among adolescents’ and parents’ perceptions of communication, family adaptability, and cohesion. Keim et al. [26] also found that age was related to problems in communication with mothers at T2 (one year later), suggesting that older age was related to more problems when communicating with mothers. However, they did not consider age as a covariate in examining the relationship between parent–child communication and child adjustment. Barrios et al. [39] invited children and adolescents aged 6–14 years old to an interview study and asked the frequently reported emotion by themselves according to the age group (younger: 6–9 years, older children: 10–14 years). They found that older children (10–14 years old, 82.1%) were more likely to have received forthright and truthful information about their illness than younger children (6–9 years old, 37%), and children who received truthful information were significantly less likely to mention fear. Finally, two studies conducted by Tillery et al. [34] and Al Ghriwati et al. [32] did not find any differences in communication practices based on participants’ age.

### 3.6. The Relationship between Parent–Child Communication and Physical Health Outcomes

There were no studies that measured the relationship between parent–child communication and physical health outcomes among the articles considered. All studies measured different types of psychological health outcomes at different levels.

### 3.7. The Relationship between Parent–Child Communication and Psychological Health Outcomes

The majority (90%) of the studies supported a positive association between parent–child communication and psychological health outcomes. The psychological health outcomes found to be beneficial included decreases in externalizing problems [32], internalizing problems, anxiety, [34], child’s anxiety level/depression, child’s withdrawal/depression scores [26], adolescent’s post-traumatic distress [8], child’s distress during port insertion [36], and child’s report of their perceived fear during treatment or hospitalization [39], and increases in the child’s self-report of their peer relationships and their quality of life [32], and enhanced social emotional strengths (self-regulation, empathy, responsibility, social competence). Table 2 provides the summary of the characteristics and behaviors associated with positive psychological outcomes.

Bai et al. [36] found that children were significantly less likely to display behavioral and verbal distress during the invasive port insertion procedure following parents’ caring verbal (e.g., avoiding assumptions, believing in self-esteem) and nonverbal communication, involving eye contact and being within a distance close enough to touch the child or the child to touch the parent. Barrios et al. [39] reported that children who received truthful information about their diagnosis were significantly less likely to mention fear during treatment or hospitalization in their qualitative design study. The importance of quality of information exchange was also emphasized [34]. They found that youths reported elevated levels of post-traumatic stress symptoms (PTSS), internalizing symptoms, and lower levels of social emotional strengths when they were struggling in maintaining desirable parent–child relationships by exchanging needed amounts of information through open and honest communication. Al Ghriwati et al. [32] examined family relationship profiles on closeness (i.e., talking about things that are supposed to be known) and discordance (i.e., disagreement with each other) and the effects on child adjustment during treatment completion. They found that children from families characterized by low closeness/high discord reported significantly greater difficulties with peer relationships compared to those who were from families with high closeness/low discord in their families.

The positive association between parent–child communication and psychological outcomes was also supported in two longitudinal design studies [8,26]. Murphy et al. [8] examined the effect of maternal PTSS on adolescents through maternal communication practice. Maternal validation mediated the negative effects of maternal PTSS on adolescent PTSS at one year. Maternal validation in communication was described as maternal behavior that acknowledges and validates the child’s verbalizations through the use of backchannels and assents. Thus, maternal validation in communication significantly reduced adolescents’ PTSS, controlling for adolescents’ PTSS at baseline.

Researchers also found some parent–child communication characteristics that were related to better family function, such as family cohesion and adaptability. For example, Philips-Salimi et al. [33] found that adolescents who perceived poor parent–adolescent communication, which is described as poor quality of communication (e.g., satisfaction with the way they communicate with each other), reported lower adolescent-perceived family adaptability, adolescent-perceived family cohesion, parent-perceived family adaptability, and parent-perceived family cohesion, after adjusting for the age and sex of the AYAs and their parents. Thus, the researchers concluded that adolescent–parent communication played a significant role in fostering a positive family environment. Similarly, Park et al. [37] found that affirming family communication was one of protective factors of family adaptation by providing children emotional support and encouragement. Greeff et al. [38] also found that affirming family communication contributed to family adaptability. Affirming family communication is characterized by taking time to hear what each other has to say or feel, working hard to ensure that family members are not emotionally or physically hurt, and respecting each other’s feelings.

Only one study did not find a significant association between parent–child communication and psychological health outcomes [35]. Viola et al. [35] evaluated the associations among problem-solving skills, parent–adolescent cancer-related communication, parent–adolescent dyadic functioning, and distress in adolescents with cancer by employing the disability–stress–coping model. They found that there was no association between cancer-related communication and adolescents’ distress.

## 4. Discussion

This review aimed to explore the characteristics of communication that contribute to positive health outcomes in the childhood cancer context and positive physical or psychological health outcomes. Our main finding was that there is preliminary evidence of a relationship between some aspects of parent–child communication and psychological health outcomes in the childhood cancer context, but there is a dearth of evidence related to physical health outcomes.

This review contributes new knowledge by identifying the specific characteristics of therapeutic parent–child communication, which contributes to better patient outcomes in the childhood cancer context. For example, although there are some existing guidelines for parents encouraging honest and open communication with children with cancer and instructions on disclosing a child’s cancer diagnoses in a safe manner, there are no specific parent–child communication guidelines that can be applied in daily life to improve patient outcomes. The additional contribution of this review to current knowledge is identifying what positive health outcomes have been studied in relation to parent–child communication. It is surprising that physical health outcomes have not been studied and presents an area of opportunity. The relationship between therapeutic interpersonal communication and positive physical health outcomes in adults with chronic illness has been widely explored. Various research studies have shown the linkage between social interaction with significant others and physiological outcomes in adult cancer survivors, which include neuroendocrine, heart, and immune functioning [41,42]; symptom distress [43,44]; and levels of stress hormones [45]. Thus, expecting a positive relationship between therapeutic parent–child communication and physical health outcomes would be reasonable. Potential physical health outcomes to explore include symptom severity such as nausea or febrile neutropenia or even treatment adherence and disease outcomes.

Most importantly, the results of this review may be meaningful to children and adolescents with cancer and their parents. In previous studies, pediatric patients with cancer and their parents reported a fear of the expected disadvantages of communication, such as hurting each other by sharing sensitive topics, as the main barrier to engagement in parent–child communication [46]. Thus, there is a need to identify the positive outcomes related to communication.

In this review, several weaknesses in parent–child communication studies were identified. Despite the evidence supporting the relationship between parent–child communication and positive psychological health outcomes, there is a lack of understanding about the mechanisms through which this occurs. For example, researchers have pointed out the need to examine the mechanism of the relationship between therapeutic family communication and coping [47,48]. Thus, future studies involving mediator, moderator analysis, or structural equation modeling around potential mechanisms are required to promote our deeper understanding.

Second, most studies focused on different characteristics and different aspects of psychological outcomes; thus, definitive conclusions are not possible. Additionally, we found that most of the studies employed cross-sectional, nonexperimental designs, with only two studies using a longitudinal design. This limitation makes identifying a causal relationship between therapeutic parent–child communication and positive health outcomes impossible.

Wide variations in terms of sample age, time since diagnosis, and diagnosis of the child with cancer were not considered in examining the association between parent–child communication and positive health outcomes. Children and adolescents require different needs in their parent–child relationship and developmental tasks according to developmental stage [49,50]. Age-related cognitive abilities would also impact communication practices with parents. In this review, 6 out of 10 articles considered age/developmental stage as a potential factor that might influence parent–child communication practices; only 2 of them reflected it in their analysis. However, the impact of age on parent–child communication practice was previously supported [8]. Future studies should either consider the influence of age on communication practices, potentially powering studies for subgroup analyses based on age or developmental stage.

In previous studies, children reported that their physical condition clearly affected their preference for being involved in parent–child communication [51]. Future studies might consider providing detailed information about the clinical characteristics of study participants or setting inclusion/exclusion criteria to minimize the influence of clinical status on parent–child communication practice or, at the very least, studies could be designed to control for these differences. Despite including studies from diverse countries, most of the population studied were White and conducted in the United States. Only one study mentioned the potential influence of culture on the relationship between parent–adolescent communication and family adaptation [37]. However, none of the remaining studies reported cultural differences and their influences on parent–child communication. There is a need to include diverse populations considering the impact of cultural background on family communication practices.

Another limitation would be that most of the included studies lack reports of parent–child communication practices from the involved participants, by including responses either from one parent or children and adolescents but failing to provide a full description of parent–child communication practices. In addition, most of the literature relied on self-report measures rather than observation. Among the literature identified, only two articles employed observation with high inter-rater reliability [8,36]. Although all studies used reliable and valid measurements, and the retrospective self-report measure is one of the most widely used methods [52], diverse data collection methods, such as observation, are required to collect objective data. Psychologists have acknowledged the importance of direct observation in assessing family communication patterns, despite some limitations [53]. In a recent topical review, Murphy et al. [54] also emphasized the objective assessment of parent–child communication practices using a reliable and unified framework by observing communication practices.

In the case of proxy reports, the majority of responses came from mothers. This might be reasonable because mothers spend the most time with their children or adolescents with cancer at their bedside or at home. Thus, mothers might be the best person to share their communication experiences. However, considering the role of fathers in parenting, the parent–child communication with fathers also needs to be assessed [55]. In addition, there is evidence showing that parent–child communication practices might be different depending on their parents’ sex [56]. In our review, only two studies included assessment from fathers’ perspectives [26,33]. Future studies should consider the inclusion of fathers’ reports to assess the triangle of communication among the child–mother–father. Additionally, considering the potential influence of parental relationships on parent–child communication [57], future studies should assess the quality of the parental relationship in studying parent–child communication. Although this review focused on parent–child communication in general nuclear families, there is also the issue of defining the parent(s) in different types of families. For example, grandparents play an important role in some extended families [58]. Additionally, it would be possible to have one parent or stepparents in divorced families. The family dynamics and parent–child communication practices might be slightly different in those families. Future research examining parent–child communication practices in the diverse forms of families is required.

Next, when employing self-report measurements, there is a need to employ age- and context-specific measurement to assess parent–child communication practices in the childhood cancer context. Although all of the studies included in this review used reliable and valid measurements to assess parent–child communication practices, we found that no measurement was age- and/or context-specific. For example, 3 out of 10 studies assessed openness/problems in parent–adolescent communication using the Parent–Adolescent Communication Scale (PACS) [26,33,39]. Despite its reliability and validity, the PACS was not specifically developed to assess parent–adolescent communication practices in the childhood cancer context. The Cancer-Related Communication Problem Scale (CRCP) was originally developed to assess whether patients and their partners have difficulty talking about cancer with each other, which had acceptable reliability in the original study [40] but presented poor reliability in the study with adolescents with cancer and their parents.

Despite the contributions of this current study, some limitations should be acknowledged. First, this study only included 10 articles and 9 out of 10 studies supported the relationship between parent–child communication and psychological health outcomes. This small sample size could not fully explain the relationship between parent–child communication and positive health outcomes. Second, this study focused on including only parent–child communication as one core aspect of family support. However, parent–child communication has multifaceted aspects that reflect the parent–child relationship, and a variety of other variables, such as development stage, also affect it. Future studies should consider the impact from other key variables in determining the relationship between parent–child communication and positive health outcomes. This study only included English-written studies. Although the included studies originated from diverse countries, such as the Republic of Korea, Spain, and Belgium, this might have caused bias by over-representing research from English-speaking countries. Finally, this study mainly focused on assessing verbal communication despite 2 out of 10 articles assessing nonverbal communication. Nonverbal communication, however, is also an important aspect in communication practices, which is mainly in charge of expressing emotions [59]. Future studies should consider including further studies assessing both verbal and nonverbal communication.

There is an emerging body of evidence that therapeutic parent–child communication contributes to better psychological health outcomes at both the individual and family levels in the childhood cancer context. The next step is to test these identified characteristics to examine the causal relationship between parent–child communication and positive health outcomes to identify the predictors of positive health outcomes. Once predictors are identified, interventions to teach those characteristics of therapeutic parent–child communication can be developed and tested.

While this review does not provide strong evidence supporting the causal relationship between therapeutic parent–child communication and positive psychological health outcomes, the evidence still supports the importance of parent–child communication in the childhood cancer context. Thus, it is important to identify children/adolescents with cancer and their families who struggle in communicating with each other and to monitor them for poor psychological health outcomes. Expressing and sharing emotions are regarded as some of the most powerful predictors in the adjustment to a cancer diagnosis [60]. Most importantly, parent–child communication does not occur in a vacuum. Thus, assessing overall parent–child relationships with a special focus on parent–child communication is recommended.

## 5. Conclusions

There is emerging evidence supporting the association between therapeutic parent–child communication and positive psychological health outcomes and the characteristics of that therapeutic communication. Future longitudinal-design studies are warranted to enhance our understanding of the causality and underlying mechanisms of the relationship between the relevant characteristics of therapeutic parent–child communication and its psychological health outcomes. Also, future study is required to explore more physical health outcomes from the engagement in therapeutic parent–child communication. The findings would contribute to the future development of parent–child communication interventions to improve psychological and physical health outcomes in the childhood cancer context. Finally, it is still important to identify children/adolescents with cancer and their families who struggle with engaging in therapeutic parent–child communication and to monitor them for poor psychological health outcomes.

## Figures and Tables

**Table 2:** Characteristics and behaviors associated with positive psychological outcomes.

Characteristics of Verbal Communication	Associated Psychological–Behavioral Outcomes
Affirming [37,38]	Better family adaptation
Open [26,33,37]	Less anxiety, less depression, better family adaptation
Satisfying [33]	Better family adjustment and cohesion
Maternal validation [8]	Lower PTSS
Avoiding assumptions [36]	Less behavioral and verbal distress during procedure
Believing what the other says [36]	Less behavioral and verbal distress during procedure
Quality of information shared [34]	Lower level of PTSS Less internalizing symptoms, Increased level of social emotional competencies
Low discord [32]	Better peer relationships, less externalizing problems
Truthful, honest communication [39]	Reduced fear
**Characteristics of Nonverbal Communication**	**Associated Psychological–Behavioral Outcomes**
Eye contact [36]	Less behavioral and verbal distress
Being physically close enough to touch [36]	More cooperative behavior during procedure
Acknowledging behavior [8]	Lower PTSS

PTSS, post-traumatic stress symptoms.

## Data Availability

All data were contained within the manuscript.
